# Plasmablastic Lymphoma Mimicking Acute Pancreatitis

**DOI:** 10.1155/2016/9751736

**Published:** 2016-03-13

**Authors:** Faisal Inayat, Hafeez Ul Hassan Virk, Ahmad R. Cheema, Muhammad Wasif Saif

**Affiliations:** ^1^Department of Medicine, New York-Presbyterian Hospital, Weill Cornell Medical College, New York, NY 10065, USA; ^2^Department of Medicine, Mount Sinai St. Luke's and Mount Sinai Roosevelt Hospitals, Icahn School of Medicine, New York, NY 10019, USA; ^3^Department of Hematology/Oncology, Tufts Medical Center, Tufts University School of Medicine, Boston, MA 02111, USA

## Abstract

*Background.* Plasmablastic lymphoma (PBL) is a rare B-cell neoplasm. It predominantly occurs in the oral cavity of human immunodeficiency virus (HIV)-positive patients and exhibits a highly aggressive clinical behavior.* Case Presentation.* We describe an unusual case of a 37-year-old HIV-positive male who presented with acute pancreatitis secondary to multiple peripancreatic masses compressing the pancreas. Histopathological examination of the lesions showed diffuse and cohesive pattern of large B-cells resembling immunoblasts or plasmablasts. The neoplastic cells were positive for BOB1 and MUM1, partially positive for CD79a, and negative for CD20, CD56, CD138, CD3, CD5, AE1/AE3, and HHV8. Epstein-Barr virus-encoded RNA in situ hybridization was positive. These features were consistent with PBL. The patient was initiated on cyclophosphamide, doxorubicin, vincristine, and prednisone (CHOP) chemotherapy, demonstrating a striking response.* Conclusion.* To our research, this is the first report of PBL with the initial presentation of acute pancreatitis. The findings in this case suggest that PBL should be included in the differential diagnosis of pancreatic and peripancreatic tumors.

## 1. Introduction

Plasmablastic lymphoma (PBL) is a rare and an almost invariably fatal clinicopathologic entity that predominantly occurs in human immunodeficiency virus (HIV)-positive patients [1]. In HIV-negative patients, PBL has been associated with older age and underlying immunosuppression secondary to transplantation, lymphoproliferative disorders, or autoimmune diseases [2]. PBL demonstrates a strong predilection for oral cavity. However, clinical spectrum of PBL has been expanding since it was first described in 1997 as a distinct entity and a subtype of diffuse large B-cell lymphoma [1]. A number of case reports in HIV-negative and multiple case series in HIV-positive patients demonstrated numerous extra-oral locations for PBL [3–12]. However, we report this rare case of PBL initially presenting with acute pancreatitis-related epigastric pain. Our patient showed a dramatic response to cyclophosphamide, doxorubicin, vincristine, and prednisone (CHOP) chemotherapy achieving near complete resolution in three months. PBL poses a formidable diagnostic challenge owing to very low incidence and associated unique morphology. It reportedly has a rapid progression with dismal outcomes resulting in death in majority of the patients within 2 years from initial presentation [13, 14]. Standard chemotherapy protocol for treatment of PBL is nonexistent.

## 2. Case Presentation

**Figure 1:**
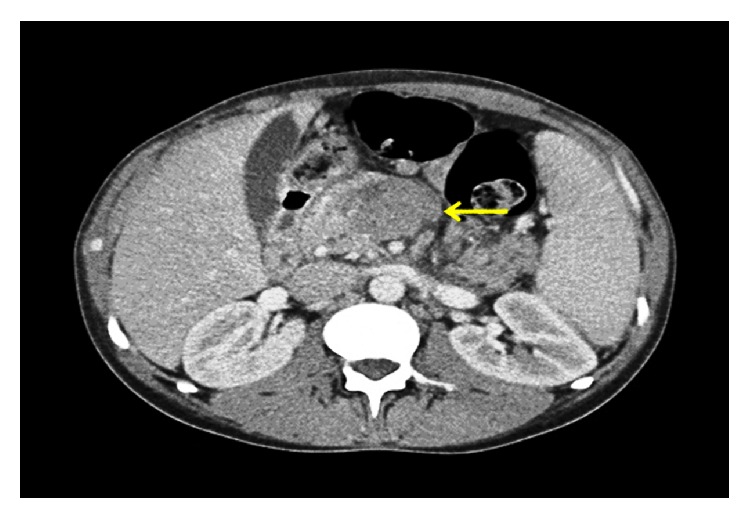
Contrast-enhanced computed tomography at the level of pancreas. Arrow demarcates multiple peripancreatic soft tissue masses to left of the uncinate process.

**Figure 2:**
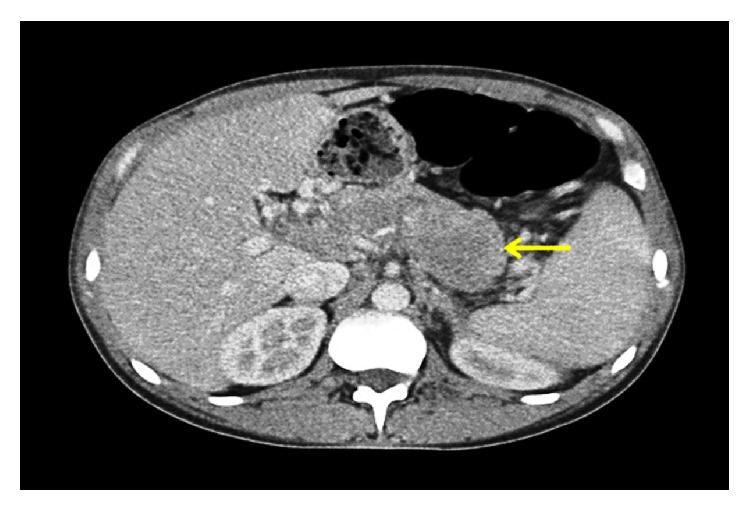
Contrast-enhanced computed tomography at the level of pancreas. Arrow demonstrates multiple peripancreatic soft tissue masses posterior to the pancreatic tail.

A 37-year-old African-American male with past medical history of HIV (detected in 2001; transmitted by heterosexual intercourse; latest CD4 count 320 cells/mm^3^ with undetectable viral load; compliant with highly active antiretroviral therapy (HAART); no opportunistic infections in past) and nephrolithiasis presented to emergency department of our hospital with progressively worsening epigastric pain and nausea for one week. Pain was constant and was rated severe and radiated to his back. The patient denied hematemesis, hematochezia, or diarrhea. However, he reported weight loss of 12 lbs in last 2 months but denied night sweats. There was no history of recent travel, sick contacts, or illicit drug use. Vital signs were notable for tachycardia of 120 beats/minute and low-grade fever of 38.8°C. The physical examination revealed epigastric tenderness with no rebound. Initial laboratory evaluation showed elevated levels of lipase 629 IU/L (33–200), amylase 250 IU/L (30–110), lactate dehydrogenase (LDH) 936 IU/L (313–618), and C-reactive protein 3.6 mg/dL (0-1), with normal liver and renal function tests. White cell count was 3.8 × 10^9^cells/L (3.8–9.8 × 10^9^), hemoglobin was 13.2 g/dL (13.5 to 17.5), and platelets were 139 × 10^9^ cells/L (150–450 × 10^9^). Cardiac troponins came out negative with unchanged EKG. Preliminary diagnosis of acute pancreatitis was made. Subsequently, abdominal computed tomography (CT) demonstrated multiple, well-defined, peripancreatic, soft tissue masses to left of the uncinate process (Figure 1), posterior to the pancreatic tail, and near the hepatic hilum (Figure 2). The peripancreatic masses were found to be compressing the body of pancreas, causing acute pancreatitis in our patient. Furthermore, there was partial compression of the portal vein with multiple surrounding portal venous system collaterals.

Differential diagnoses included lymphoma, mycobacterium avium complex (MAC), disseminated fungal infection, and tuberculosis (TB). The case was discussed in the multidisciplinary tumor board. It was concluded that, due to the advanced disease, tissue diagnosis will be difficult employing surgery or interventional radiology. Thereafter, an uneventful endoscopic ultrasound (EUS)-guided fine-needle aspiration biopsy of one of the masses was performed, and preliminary pathology suggested lymphoma. Cancer antigen 19-9 (CA 19-9), carcinoembryonic antigen (CEA), alpha-fetoprotein (AFP), and serotonin levels were within normal limits. Sputum AFB culture and QuantiFERON-TB Gold (QFT) were negative ruling out TB. Fungal cultures and isolated bacterial cultures for MAC also came out negative. Total body scan showed subcentimeter bilateral mediastinal and axillary lymphadenopathy. Histopathology analysis revealed diffused infiltration of large B-cells with plasmablastic (cells with rounded nuclei, coarser chromatin, and smaller two to three nucleoli) and immunoblastic morphological features (cells with vesicular enlarged nuclei and single prominent nucleolus). A high proliferative index with frequent apoptosis and focal necrosis was demonstrated. Immunohistochemistry of the peripancreatic specimen revealed neoplastic cells positive for BOB1, MUM1, and EBER-ISH, partially positive for CD79a, and negative for CD20, CD56, CD138, CD3, CD5, AE1/AE3, and HHV8, most consistent with plasmablastic lymphoma. A bone marrow biopsy was performed for staging purposes. It revealed normocellular marrow with maturing trilineage hematopoiesis with no evidence of excess blasts or lymphoma. Furthermore, lumber puncture cytology was negative for malignant cells and CSF flow cytometry did not show any abnormal cells; based on these findings, the patient was deemed as stage III.

**Figure 3:**
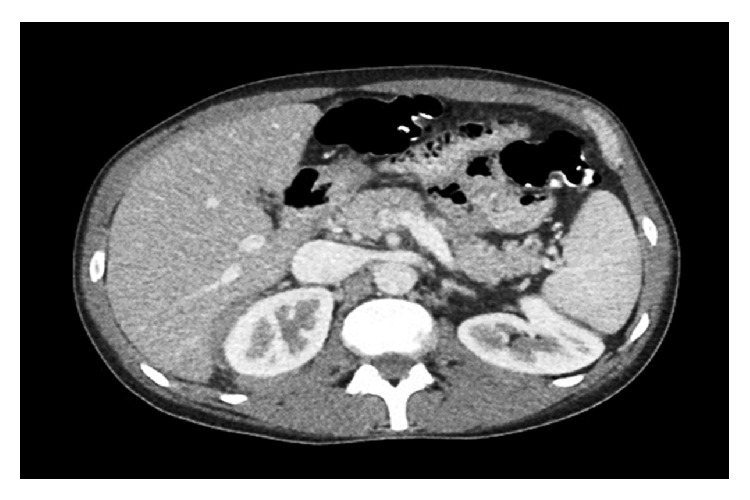
Contrast-enhanced computed tomography at the level of pancreas after 3 months of cyclophosphamide, doxorubicin, vincristine, and prednisone (CHOP) treatment demonstrating near complete resolution of previously seen soft tissue masses.

Subsequently, epigastric pain improved with oral opioids and other pancreatitis-related symptoms resolved with the symptomatic treatment. The patient was discharged from the hospital with oncology out-patient follow-up. Therein, he was initiated on cyclophosphamide, doxorubicin, vincristine, and prednisone (CHOP) therapy. Central nervous system prophylaxis was achieved by employing intrathecal chemotherapy. Filgrastim (G-CSF) was administered twenty-four hours after CHOP cycles and was continued daily for seven days. Our patient tolerated the treatment well, with no significant interactions with HAART for his HIV-infection, and achieved remission after six cycles of chemotherapy. Contrast-enhanced CT at the level of pancreas 3 months after initiation of CHOP treatment demonstrated near complete resolution of previously seen soft tissue masses (Figure 3). The patient has been disease-free for over a year now without any additional specific therapy.

## 3. Discussion

Plasmablastic lymphoma (PBL) is a distinct, diffuse large B-cell lymphoma, commonly associated with HIV-infection [1]. It accounts for 1.5% of all nodal non-Hodgkin's lymphomas and 2.6% of all AIDS-related neoplasms [2]. PBL typically involves oral sites in HIV-positive population [2]. However, newer reports demonstrated PBL in numerous extra-oral locations predominantly gastrointestinal tract, lymph nodes, skin, bone, genitourinary, nose, central nervous system, liver, and lung, with or without HIV-infection [3–12]. Previously, pancreas or peripancreatic tissue involvement has rarely been described. To the best of our knowledge, this is the first case of PBL with initial presentation of acute pancreatitis.

PBL is associated with Epstein-Barr virus (EBV) infection in over 75% of the HIV-positive cases [11]. Chronic EBV infection thrives in the environment generated by HIV and EBV inhibits apoptosis in B-cells by mechanisms related to EBV antigens, unlocking their malignant potential. Hence, it plays a significant role in the tumorigenesis in HIV-positive PBL patients [2, 11–14]. Apart from the role of EBV as an effective diagnostic marker, it has also been implicated in investigating potential EBV-directed PBL therapies and predicting treatment response and relapse in PBL cases [15–17].

PBL represents a diagnostic and therapeutic challenge for pathologists and clinicians alike, owing to scarcity of the available literature and indistinguishable tumor cells from plasmablastic myeloma [18]. It is a high-grade neoplasm with diffuse lymphoid infiltrates and cohesive growth pattern, having cytomorphologic features such as large B-immunoblasts with an immunophenotype of plasma cells [18]. PBL cells are immunohistologically positive for CD79a, IRF-4/MUM-1, BLIMP-1, CD38, and CD138; negative for B-cell markers CD19, CD20, and PAX-5 and some cases express T-cell marker CD2 or CD4 [19]. Immunohistochemistry with antibody MIB-1 revealed that most or all neoplastic cells are positive for Ki-67 and negative for HHV8. 50% of the PBL cases express an* MYC* translocation indicatingpoor prognosis [20]. EBV-encoded RNA in situ hybridization (EBER-ISH) is a very sensitive modality that is usually implicated in HIV-positive PBL patients with oral locations [21]. In our case, tumor cells were positive for BOB1, MUM1, and EBER-ISH, partially positive for CD79a, and negative for CD20, CD56, CD138, CD3, CD5, AE1/AE3, and HHV8, which was most consistent with plasmablastic lymphoma. Therefore, clinicians and pathologists should have this location in mind for plasmablastic lymphoma.

Clinically, PBL is a very aggressive lymphoma. Two-year overall survival rate for PBL patients was 43% in a recent study conducted in German [13]. Increased mortality in PBL patients is due to pathologic misdiagnosis, delayed initial presentation at an advanced stage (III or IV), and lack of optimal therapy [2]. Over 55% of PBL patients are at stage IV at the time of diagnosis in extra-oral as compared to oral sites, indicating more common dissemination in extra-oral PBL [18]. In our case, the primary PBL of the peripancreatic tissues had infiltrated adjacent lymph nodes, and resulting lymphadenopathy compressed the pancreas; accordingly a clinical stage III was assigned. Our patient demonstrated a dramatic response to CHOP treatment. However, standard therapeutic approach for PBL patients has not been reached so far. Previously employed chemotherapy regimens include cyclophosphamide, doxorubicin, vincristine, and prednisone (CHOP); dose-adjusted etoposide, prednisone, vincristine, cyclophosphamide, and doxorubicin (DA-EPOCH); and cyclophosphamide, vincristine, doxorubicin, high-dose methotrexate/ifosfamide, etoposide, and high-dose cytarabine (CODOX-M/IVAC) [22]. Recently, bortezomib and dose-adjusted EPOCH have demonstrated efficacy in untreated patients with HIV-positive PBL [23]. Future studies aiming at EBV antigens or/and* MYC* dysregulation in addition to autologous stem cell transplant may help to devise an effective treatment approach for PBL.

## 4. Conclusion

This paper implicates that plasmablastic lymphoma, albeit rare, should be included in the differential diagnosis of pancreatic and peripancreatic tumors. The diagnosis can be challenging due to complex cytomorphologic and immunophenotypic characteristics, particularly in extra-oral locations. Plasmablastic lymphoma follows an aggressive clinical course and associated with poor outcomes. Hence, the clinicians and pathologists should always maintain a high index of suspicion for this hard-to-diagnose and hard-to-treat disease.
